# The complete chloroplast genome sequence of *Acorus gramineus* (Acoraceae)

**DOI:** 10.1080/23802359.2019.1677182

**Published:** 2020-01-10

**Authors:** Xingfu Zhu, Xiaoxin Tang, Yin Yi

**Affiliations:** a The State Key Laboratory of Southwest Karst Mountain Biodiversity Conservation of National Forestry and Grassland Administration, College of Life Science, Guizhou Normal University, Guiyang, China;; b The Key Laboratory of Plant Physiology and Development in Guizhou Province, Guizhou Normal University, Guiyang, China

**Keywords:** Chloroplast, *Acorus gramineus*, phylogenetic analysis

## Abstract

The complete chloroplast genome sequence of *Acorus gramineus* was assembled and characterized as a resource for future genetic studies. With a total length of 152,887 bp, the chloroplast genome comprised of a large single-copy (LSC) region of 83,005 bp, a small single-copy (SSC) region of 18,230 bp, and two inverted repeat (IR) regions of 25,826 bp. The overall GC contents of the chloroplast genome were 38.7%. A total of 115 genes were predicted, consisting of 80 protein-coding genes, 31 tRNA genes, and 4 rRNA genes. In these genes, nine genes contained one intron and two genes contained two introns. Phylogenetic analysis confirmed the position of *A. gramineus* within the monocots.

*Acorus* is a genus of monocot flowering plants in the family Acoraceae and order Acorales. They are found in wetlands, particularly marshes, where they spread by means of thick rhizomes. Modern phylogenetic studies demonstrated that *Acorus* is the most primitive of the monocotyledons still in existence (Goremykin et al. 2005). Consequently, the genetic and genomic information is important to determine the phylogenetic relationships among the major lines of angiosperms. Here, we report and characterize the complete plastome of *A. gramineus* for future genetic studies on related species (GenBank accession number: MN175740).

The total genomic DNA was extracted from dry leaves sampled from Kunming (Yunnan, China, E 102.5848°, N 24.5773°, 1994 m a.s.l.) and a voucher herbarium specimen (Accession number: Zhu201904002) was deposited at the Herbarium of Guizhou Normal University. Total genomic DNA was extracted with the Qiagen DNeasy Plant Mini Kit (Qiagen, Carlsbad, CA, USA). The genomic paired-end (PE150) sequencing was performed on an Illumina Hiseq 2000 instrument. The cp genome was assembled using the program NOVOPlasty (Dierckxsens et al. 2017). Annotation was performed using PGA (Qu et al. 2019), coupled with manual correction for start and stop codons of protein-coding genes.

The complete chloroplast genome of *A. gramineus* is 152,887 base pairs (bp) in length and contains a large single-copy region of 83,005 bp, a small single-copy region of 18,230 bp, and two inverted repeat regions of 25,826 bp. The overall GC content was 38.7%, while higher in IR (42.84%) than in LSC (37.3%) and SSC (33.2%). The plastome comprised 115 unique genes in total, including 80 protein-coding genes (PCGs), 31 tRNAs and 4 rRNAs. In these gens, nine genes (ndhA, ndhB, petB, petD, rpl2, rpl16, rpoC1, rps12, rps16) have one intron, and two genes (ycf3 and clpP) have two introns. Six protein-coding genes, eight tRNAs and all four rRNAs were completely duplicated within IRs.

To further investigate its phylogenetic position, a maximum likelihood tree was constructed based on complete chloroplast genome sequences of ten other monocotyledonous species and one dicotyledonous species using RAxML (Stamatakis 2014) after the sequences were aligned using MAFFT v7.307 (Katoh and Standley 2013). Our results suggested the clade including *A. gramineus* and *A. americanus* is sister to all other monocots (Figure 1). This published *A. gramineus* chloroplast genome will provide useful information for the relationships among the major lines of angiosperms.

**Figure 1.**
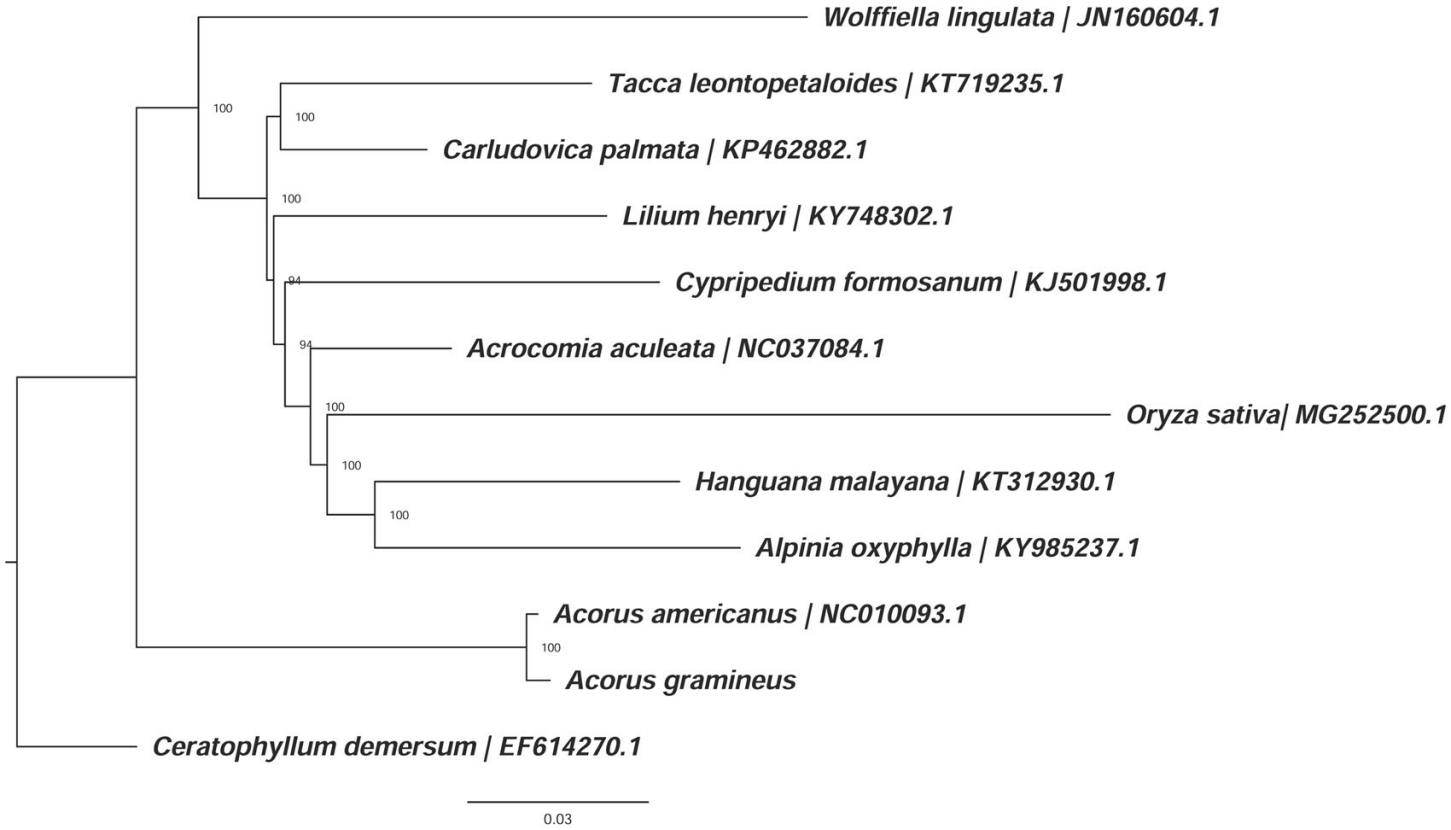
ML phylogenetic tree of elven species within monocotyledons based on ten chloroplast genome sequences in GenBank, plus the chloroplast sequence of *Acorus gramineus*. The tree is rooted with the dicotyledons (*Ceratophyllum demersum)*. Bootstraps (10,000 replicates) are shown at the nodes.
